# Shifts in Rhizosphere and Bulk Soil Microbial Communities During the Second and Third Years of Ginseng Cultivation

**DOI:** 10.3390/microorganisms14040764

**Published:** 2026-03-27

**Authors:** Deqiang Yang, Zhipeng Xu, Ruitong Du, Yunwei Liu, Xiangquan Li, Zhibin Wang

**Affiliations:** 1 Key Laboratory of Basic and Application Research of Beiyao, Ministry of Education, Heilongjiang University of Chinese Medicine, Harbin 150040, China; 2 Yichun Branch of Heilongjiang Academy of Forestry Sciences, Yichun 153000, China

**Keywords:** rhizosphere soil, original soil, soil bacteria, soil fungi

## Abstract

Soil microbial communities regulate plant growth and nutrient cycling, yet their dynamics during early ginseng cultivation remain poorly understood. This study used Illumina MiSeq sequencing to characterize bacterial and fungal communities in rhizosphere and bulk soils from second- and third-year ginseng fields. Differences across growth periods were analyzed using one-way ANOVA. Significant shifts in α- and β-diversity occurred in both soil rhizosphere and bulk soils, and distinct environmental factors shaped community structure. Correlation heatmaps, redundancy analysis (RDA), and Mantel tests identified associations between soil physicochemical properties and microbial taxa. Notably, soil location had a stronger effect on microbial variation than cultivation duration. Dominant bacterial genera were strongly correlated with NH_4_^+^-N, and fungal community composition was primarily driven by NH_4_^+^-N. These results demonstrate that early ginseng cultivation significantly alters soil microbial communities and provide a basis for sustainable agricultural practices and soil ecosystem management.

## 1. Introduction

Soil microorganisms are key regulators of soil ecosystem functioning. They influence plant growth, nutrient cycling, and overall soil health [1]. The rhizosphere—the narrow soil zone is directly affected by root exudates and root-associated biological activity—differs markedly from bulk non-rhizosphere) soil, which represents the undisturbed background soil [2]. Bulk soil retains its native physicochemical properties and indigenous microbial communities and therefore serves as a reference for natural soil ecology [3,4]. In contrast, the rhizosphere is characterized by steep biogeochemical gradients and high microbial metabolic activity [5,6]. These conditions promote the selective enrichment of plant-beneficial microorganisms, including nitrogen-fixing bacteria phosphate-solubilizing microbes, and plant growth promoting rhizobacteria, while suppressing potential pathogens. Although bulk soil generally exhibits higher phylogenetic diversity and greater functional, its microbial communities engage in fewer direct plant-mediated interactions [7,8,9]. The differences in microbial composition, activity, and functional potential between rhizosphere and bulk soil collectively regulate soil fertility, biogeochemical processes, disease suppression, and plant performance, reflecting the hierarchical organization and functional specialization of soil ecosystems.

*Panax ginseng* C.A. Meyer is a widely valued medicinal herb with a long history of use in traditional medicine. Its pharmacological effects are largely attributed to bioactive compounds such as ginsenosides, polysaccharides, and amino acids, which confer antioxidants, metabolic regulatory, cardioprotective, and neuroregulatory activities. Two main cultivation systems are currently used: eco-forest cultivation, which simulates natural growth conditions, and standardized field cultivation. Although forest-based cultivation better preserves medicinal quality, it requires a long growth cycle and cannot meet increasing market demand. In commercial production systems, ginseng is typically harvested after four to six years, with the two- to three-year seedling stage being particularly critical for plant development. A major constraint in ginseng cultivation is its intolerance to continuous cropping; soils previously used for ginseng often become unsuitable for replanting. Prolonged cultivation also reduces microbial abundance and alters soil microbial structure. Moreover, ginsenosides such as Rb1 and Rd can stimulate *Fusarium* spore germination at low concentrations, increasing the risk of root rot and further threatening sustainable production [10,11,12].

Previous studies show that soil microbial communities in the ginseng rhizosphere and bulk soil change dynamically across cultivation years [10,11]. For instance, in the rhizosphere, microbial diversity and activity generally increase over time with beneficial plant growth promoting taxa significantly enriched by the third year [12]. These trends highlight the role of root exudates in shaping microbial community structure [13]. In contrast, microbial shifts in bulk soil are less pronounced [14,15]. Continuous cropping of *Panax ginseng* leads to soil acidification and increased soil-borne diseases (e.g., root rot), often necessitating fallow periods of up to 30 years before replanting. Cultivation age also influences rhizosphere composition. In four-year-old ginseng, the rhizosphere is enriched with degradative bacteria such as *Sphingomonas* and beneficial fungi such as *Tetracladium*, which are positively correlated with yield. Although overall fungal abundance declines by 57.5% over six years, pathogen-suppressive genera (e.g., *Exophiala*, *Humicola*) increase in the rhizosphere [1,10]. Despite these advances, a critical knowledge gap persists: no targeted investigations have yet characterized the soil microbiome throughout the 2–3-year juvenile phase of ginseng cultivation, which is essential for seedling establishment and the initial assembly of the rhizosphere microbiome before harvest. Existing studies (e.g., Jin et al., 2022) [12] primarily examined broad pre- and post-cultivation shifts across farmlands of different ages without compartment-specific (rhizosphere vs. bulk soil) analysis during this early stage. Similarly, Tong et al. (2021) [16] and Lan et al. (2023) [17] aggregate data across cultivation modes and years, overlooking year-specific juvenile dynamics and physicochemical–microbial interactions. Therefore the 2–3-year juvenile stage of cultivated ginseng remains insufficiently understood.

The 2–3-year cultivation period represents a critical physiological and ecological transition in ginseng. During this juvenile stage, rapid root expansion and increased root exudation substantially modify the soil microenvironment. This phase marks the initial divergence between rhizosphere and bulk soil microbial communities and the early accumulation of beneficial or pathogenic taxa before visible disease symptoms emerge. Although long-term chrono sequence studies (e.g., Shi et al., 2024) [18] described broad successional trends, the precise high-resolution spatial (rhizosphere vs. bulk soil) and short-term temporal (year 2 to year 3) microecological dynamics during this early-warning stage remain poorly understood.

To address this knowledge gap, we investigated the soil microecological dynamics of 2- and 3-year-old farmland-cultivated ginseng by using high-throughput sequencing. We aim to: (1) characterize the changes in both diversity and community composition of bacterial and fungal communities across rhizosphere and bulk soils; and (2) identify the key soil physicochemical parameters driving the soil microbiome. Our hypotheses: (i) The continuous cropping of ginseng over two to three years would lead to a distinct structural divergence in microbial diversity and community composition between the rhizosphere and bulk soil compartments. (ii) Cultivation-induced alterations in soil physicochemical gradients (such as shifts in pH and available nutrients) would act as the primary deterministic factors shaping microbial community structures, leading to the selective enrichment of specific microbial taxa adapted to the dynamic rhizosphere microenvironment.

## 2. Materials and Methods

### 2.1. Study Sites

This study located in the Tieli City, Heilongjiang Province (N: 47°09′19.38″, E: 128°15′ 50.30″). The climate is a typical temperate continental monsoon, with four distinct seasons. The average annual temperature is about 2.4 °C, with maximum temperatures reaching 31.6 °C and minimum temperatures −38.8 °C. The study included soil samples from five ginseng cultivation conditions: two-year ginseng bulk soil (PGOC2) and rhizosphere soil (PGRC2), three-year ginseng bulk soil (PGOC3) and rhizosphere soil (PGRC3), along with control soil without ginseng cultivation (PGC). Ginseng seedlings were established by sowing seeds in dedicated seedbeds before being transplanted to raised beds with a width of 150–170 cm. These beds were filled with loose, highly permeable, and well-drained soil. Before transplanting, basal fertilization was implemented by applying 5–10 kg of farmyard manure and approximately 50 g of superphosphate or compound fertilizer per square meter into the ditches, followed by soil covering. The relative soil moisture content was maintained at approximately 20% throughout the cultivation period. For each of the five treatments (PGC, PGOC2, PGRC2, PGOC3, PGRC3), three independent replicate plots (10 m × 10 m) were established. Within each plot, we employed a standard five-point sampling strategy. To obtain the bulk soil, five soil cores were collected and thoroughly mixed to form one composite bulk soil sample per plot. For the rhizosphere soil, 5 healthy ginseng plants with uniform growth were carefully uprooted from each plot. The rhizosphere soils brushed from these 5 plants were pooled together to form one composite rhizosphere sample per plot. All PGOC2 and 3 and PGRC2 and 3 samples were placed in labeled sterile bags and sealed immediately after collection. Samples were transported to the laboratory under low-temperature conditions in biological sample boxes prepacked with dry ice. Upon arrival, soils passed through a 2 mm sieve to remove stones, visible roots, and debris. Each bulk soil and rhizosphere soil sample was then divided into two portions: one stored at 4 °C for physicochemical analysis, and the other preserved at −80 °C for subsequent microbial analysis.

### 2.2. Soil Chemical Properties

The following analytical methods were employed to characterize the soil properties. Soil pH was measured with a pH meter (Thermo Scientific Orion 3-Star Benchtop, Leicester, UK) using a 1:2.5 (*w*/*v*) soil-water suspension after 30 min of shaking [19]. Soil organic matter (SOM) content was determined by the potassium dichromate oxidation method with external heating [20]. For nutrient analysis, total carbon (TC) and total nitrogen (TN) were quantified using a Vario Max CNS elemental analyzer (Elementar, Langenselbold, Germany) [21]. The concentrations of inorganic nitrogen, namely nitrate nitrogen (NO_3_^−^-N) and ammonium nitrogen (NH_4_^+^-N), were assessed with a continuous flow analyzer [22]. Phosphorus content was analyzed using two approaches: total phosphorus (TP) was measured by Inductively Coupled Plasma Optical Emission Spectrometry (Agilent 5100 ICP-OES, Melbourne, Australia) [23], while available phosphorus (AP) was extracted with 0.5 M NaHCO_3_ and subsequently determined by the Mo-Sb colorimetric method [19,23]. Finally, soil water content (SWC) was ascertained by the standard oven-drying technique [24].

### 2.3. DNA Extraction and High-Throughput Sequencing

The extraction of soil total DNA was carried out with the E.Z.N.A.^®^ DNA Kit (OmegaBio-tek, Norcross, GA, USA). The quality and purity of DNA were assessed using a NanoDrop 2000 (NanoDrop Technologies, Wilmington, DE, USA), and further visualized through electrophoresis on 1% agarose gels. The primers used for bacteria detection were 515F (5′-GTGCCAGCMGCCGCGG-3′) paired with 806R (5′-GGACTACHVGGGTWTCTAAT-3′), while for fungi detection, the primers were ITS1F (5′-CTTGGTCATTTAGAGGAAGTAA-3′) and ITS2R (5′-GCTGCGTTCTTCATCGATGC-3′) [25,26]. PCR products were recovered and refined by means of a 2% agarose gel along with Axy Prep DNA Gel Extraction Kits (Axygen Biosciences, Union City, CA, USA). Subsequently, sequencing was carried out on an Illumina MiSeq platform, facilitated by Shanghai Majorbio Bio-pharm Technology Co., Ltd. (Shanghai, China). The Illumina MiSeq-PE300 platform (Illumina, San Diego, CA, USA) was successfully used to complete all sequencing. QIIME 2 was employed for microbiome bioinformatics analysis [27]. The demux plugin was utilized to demultiplex the raw paired end reads, followed by the removal of primers using the cut adapt plugin [28]. Subsequently, the sequences underwent quality filtering and denoising through the Dada2 process as per previous studies. To ensure sequence quality and eliminate all sequencing errors, we applied the learnErrors, derepFastq, dada, and mergePairs methods with their default settings. Following this, the amplicon sequence variants (ASVs) representing single occurrences (singletons) were removed. Subsequently, the Silva v132 database (http://www.arb-silva.de) (accessed on 8 September 2025) [29] was used to determine the taxonomic identity of bacteria, and the UNITE database [30] was used to determine the taxonomic identity of fungi. To avoid analytical biases caused by variations in sequencing depth, the ASV tables for both bacterial and fungal communities were rarefied (normalized) to an identical sequencing depth of 0.999 reads per sample based on the sample with the lowest sequence count. Subsequent alpha- and beta-diversity calculations were performed based on these normalized ASV tables. Furthermore, Good’s coverage was calculated to estimate the completeness of sequencing.

### 2.4. Statistical Analysis

Soil physical and chemical properties were analyzed by one-way ANOVA and *t*-test [31]. The non-metric multidimensional scaling (NMDS) analysis, a powerful tool for visualizing and interpreting complex multivariate data, was conducted by employing the renowned “ggplot2” and “vegan” packages within the versatile R software (4.5.1) environment [32]. The stacked plots, which provide a visual representation of multiple variables across different categories, were carefully constructed using the “ggplot2” package in the R software [33]. The metacoder taxonomy tree was drawn by using the “metacoder” package in the R software [34]. We performed Kruskal–Wallis analysis with the help of the “stats” package in the R software [35]. We plotted Correlation heatmap with the help of “corrmorant”, “pheatmap”, “corrplot” packages in the R software [36]. Mantel test was performed with the assistance of the “vegan” and “ggcor” packages in the R software [37].

## 3. Results

### 3.1. Soil Physical and Chemical Properties

Soil physicochemical properties differed significantly among the five treatments. The PGOC2 treatment produced the lowest pH and the highest available phosphorus (AP) and total phosphorus (TP) levels. The ammonium nitrogen (NH_4_^+^-N) content was highest under PGOC3, whereas nitrate nitrogen (NO_3_^−^-N) was highest under PGRC3. In contrast, soil organic matter (SOM) and total nitrogen (TN) did not differ significantly among treatments. (Table S1).

### 3.2. Soil Bacterial and Fungal Community Diversity

High-throughput sequencing of the 15 soil samples generated raw reads targeting the bacterial 16S rRNA and fungal ITS regions. After quality filtering and chimera removal, 318,330 high-quality bacterial reads (averaging 21,222 per sample) and 484,755 high-quality fungal reads (averaging 32,317 per sample) were retained for downstream analysis. Good’s coverage exceeded 0.999 for all samples, and rarefaction curves (Supplementary Figure S1) plateaued, indicating sufficient sequencing depth to capture microbial diversity in both the rhizosphere and bulk soils.

**Figure 1:**
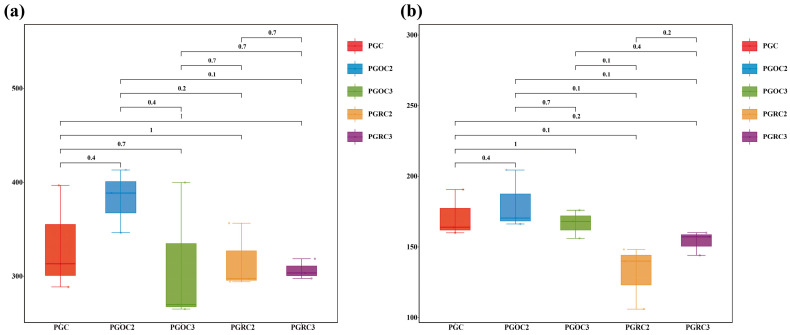
The richness (Chao1 index) of soil bacterial (**a**) and fungal communities (**b**) in different treatments. Different numbers between groups indicated significant differences in soil bacterial communities under different treatments at the 5% level (*p* < 0.05). Statistically significant differences among soil depth were analyzed using ANOVA and Tukey’s post hoc test. (The dots of the same color represent the samples in the same group.).

**Figure 2:**
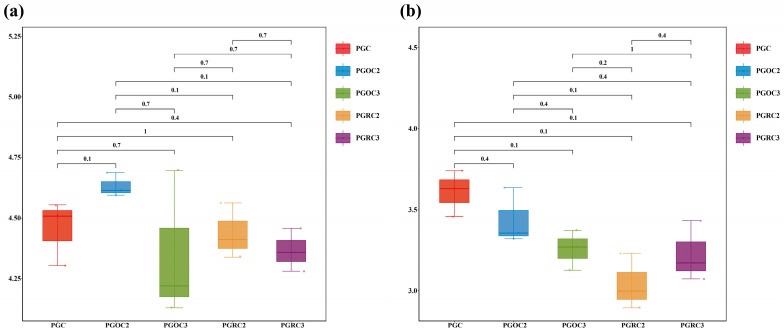
The diversity (Shannon index) of soil bacterial (**a**) and fungal communities (**b**) in different treatments. Different numbers between groups indicated significant differences in soil bacterial communities under different treatments at the 5% level (*p* < 0.05). Statistically significant differences among soil depth were analyzed using ANOVA and Tukey’s post hoc test. (The dots of the same color represent the samples in the same group.).

Our study demonstrated that the alpha-diversity of bacterial and fungal communities varied among treatments (Tables S2 and S3). The Simpson index showed no significant differences, whereas ACE, Chao1, and Shannon indices differed significantly (*p* < 0.05). In non-rhizosphere soils, all alpha diversity indices varied significantly among cultivation years (Figure 1, *p* < 0.05). Shannon and ACE indices followed the order: PGOC2 > PGC > PGOC3 > PGRC3 > PGRC2, with Chao1 showing a similar trend. In rhizosphere soils, significant differences in fungal alpha diversity were detected only between the second and third cultivation years (Figure 2, *p* < 0.05). Bacterial Shannon and Simpson indices did not differ significantly among treatments. Overall, cultivation exerted a stronger effect on microbial diversity in non-rhizosphere soils than in rhizosphere soils.

**Figure 3:**
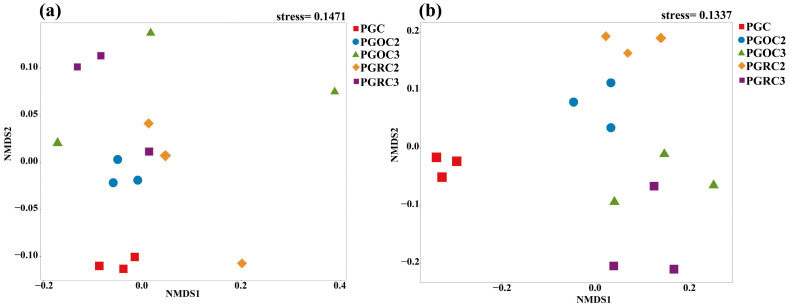
Non-metric multidimensional scaling analysis (NMDS) demonstrated the different compositional relationships among soil bacterial (**a**) and fungal communities (**b**) under different treatments. Each point in the figure represents a sample, and the distance between the points reflects the similarity or difference between the samples. Closer points indicate that the samples are more similar to each other, while points that are further apart indicate greater differences between the samples. Different colors represent different treatments.

Non-metric multidimensional scaling (NMDS) revealed clear separation of soil bacterial and fungal communities under the PGC treatment from the other four treatments. Soil bacterial communities showed high similarity among PGOC2, PGRC2, and PGRC3 treatments. Soil fungal communities were highly similar between PGOC2 and PGRC2, and between PGOC3 and PGRC3. Notably, soil fungal communities in rhizosphere and non-rhizosphere soil within the same year exhibited strong similarity (Figure 3). PERMANOVA confirmed a significant treatment effect on bacterial community composition (R^2^ = 0.42, F = 8.76, *p* < 0.05), indicating that treatment explained 42% of the variation. A significant effect was also observed for fungal communities (R^2^ = 0.38, F = 7.24, *p* < 0.05), accounting for 38% of the compositional variance. These results support the clustering patterns observed in the NMDS analysis: PGC samples were distinctly separated from all ginseng-cultivated soils, while rhizosphere (PGRC) and bulk (PGOC) soils from the same year showed greater similarity than soils from different years, particularly for fungi (Figure 3b).

### 3.3. Soil Bacterial and Fungal Community Composition

**Figure 4:**
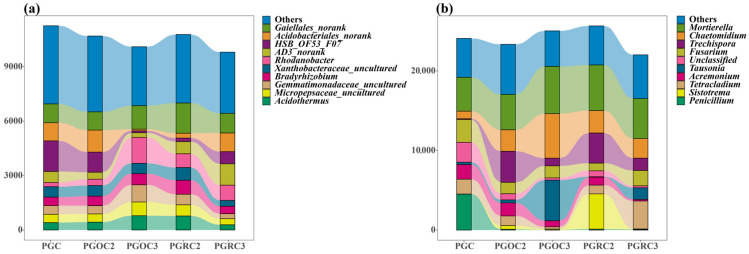
The stacked plots show the ranking of the soil bacterial (**a**) and fungal (**b**) genera under different treatments. Different genres of bacteria and fungi are distinguished by blocks of different colors, each color representing a specific category.

Stacked bar plots at the genus level further illustrated treatment-specific compositional differences (Figure 4). In bacterial communities (Figure 4a), *HSB_OF53_FO7* was more abundant under PGC and PGOC2 than in the other treatments, whereas *Rhodanobacter* peaked under PGOC3. In contrast, *Gaiellales*_norank remained relatively stable across treatments. In fungal communities (Figure 4b), *Mortierella* showed consistent abundance across all treatments, while *Chaetomidium* and *Tausonia* were most abundant under PGOC3. Overall, these treatment-driven shifts clarify how different cultivation conditions influence soil microbiota.

From the second to the third cultivation year, soil microbial communities exhibited clear temporal shifts. In rhizosphere soil, the total abundance of both bacteria and fungi declined in year 3 compared with year 2. Among dominant bacterial genera (relative abundance > 1%), *Gaiellales*_norank, uncultured *Xanthobacteraceae*, uncultured *Gemmatimonadaceae*, uncultured *Micropepsaceae*, *Bradyrhizobium*, and *Acidothermus* decreased, whereas *Acidobacteriales*_norank, *HSB_OF53_FO7*, and *AD3*_norank increased. Fungal communities showed a similar overall decline, with only a few genera *Fusarium*, *Tausonia*, and *Tetracladium*—increasing in relative abundance. In non-rhizosphere soil, bacterial abundance generally decreased from year 2 to year 3. Notable declines were observed in *HSB_OF53_FO7* and *Acidobacteriales*_norank, whereas *Gaiellales*_norank, *Rhodanobacter*, uncultured *Gemmatimonadaceae*, uncultured *Micropepsaceae*, and *Acidothermus* increased. In contrast, fungal abundance increased in year 3. Except for *Trechispora*, *Acremonium*, *Tetracladium*, and *Sistotrema*, most fungal genera showed enrichment. These findings indicate distinct responses of rhizosphere and non-rhizosphere communities to cultivation duration, with bacteria and fungi displaying opposite successional trends in non-rhizosphere soil.

**Figure 5:**
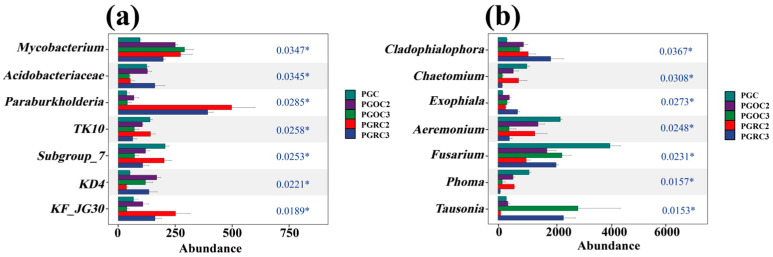
The Kruskal–Wallis test was used to determine the differences between the soil bacterial (**a**) and fungal (**b**) genera in different treatments. (‘*’ indicates *p* < 0.05). The blue numbers (*p*-values) on the right side of the graph are used to determine if there is a significant difference between the groups of data.

The Kruskal–Wallis test, a non-parametric method that does not assume a specific distribution, showed that Mycobacterium abundance did not differ significantly among PGOC2, PGOC3, PGRC2, and PGRC3 treatments. *Paraburkholderia* exhibited the highest abundance under PGRC2 and PGRC3. TK10 and Subgroup_7 were more abundant under PGC and PGRC2 than under the other treatments (Figure 5a). For fungi, *Tausonia* was more abundant under PGOC3 and PGRC3, whereas *Acremonium* and *Fusarium* were most abundant under PGC (Figure 5b).

### 3.4. Factors Affecting Soil Microbial Communities

**Figure 6:**
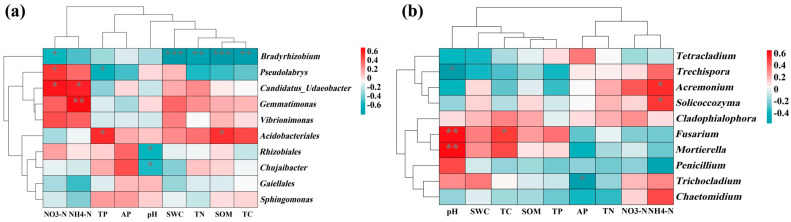
Correlation heatmap showed the correlation between soil physico−chemical properties and the top ranked soil bacterial (**a**) and fungal (**b**) genera, respectively, under different treatments. Red represents positive correlation and blue represents negative correlation. (‘*’ indicates *p* < 0.05; ‘**’ indicates *p* < 0.01; ‘***’ indicates *p* < 0.001). The correlation between different variables was derived based on the Pearson correlation coefficient (Pearson correlation coefficient).

Among the dominant bacterial genera, *Bradyrhizobium* was negatively correlated with soil total carbon (TC, SOM, TN and soil water content SWC). *Acidobacteriales* showed a positive correlation with SOM and TP. *Rhizobiales* and *Chujaibacter* were negatively correlated with pH (Figure 6a). Among dominant fungal genera, *Trechispora* was negatively correlated with pH, whereas *Fusarium* and *Mortierella* were positively correlated with pH. *Acremonium* and *Solicoccozyma* were positively correlated with NH_4_^+^-N (Figure 6b).

**Figure 7:**
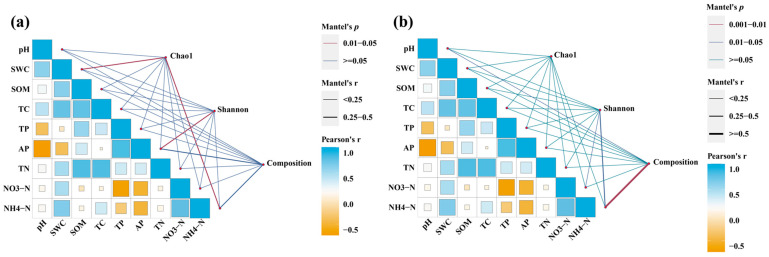
Mantel test showed the potential drivers of soil bacterial (**a**) and fungal communities (**b**). Edge width corresponds to Mantel’s r value, and the edge color denotes the statistical significance. Pairwise correlations of these variables are shown with a color gradient presenting Pearson’s correlation coefficient.

Mantel analysis showed that the bacterial Chao1 index was significantly correlated with soil SWC and NH_4_^+^-N (*p* < 0.05, whereas the bacterial Shannon index was significantly correlated with soil AP (*p* < 0.05) (Figure 7a). For fungal communities, the Shannon index was significantly correlated with NH_4_^+^-N (*p* < 0.05). Fungal community composition was also significantly associated with NH_4_^+^-N (Figure 7b).

Redundancy analysis (RDA) explained 88.37% and 81.38% of the total variation in bacterial and fungal community composition, respectively. For rhizosphere bacteria, PGC and PGCO3 showed similar responses to soil properties, with positive correlations with SWC and pH and negative correlations with AP and TP. In contrast, PGCO2 was positively correlated with AP and TP but negatively correlated with SWC and pH. PGRC3 was positively associated with total carbon (TC), NH_4_^+^-N and soil organic matter (SOM) and negatively associated with NO_3_^−^-N. PGRC2 showed positive correlations with AP and NO_3_^−^-N and negative correlations with TC, total nitrogen (TN), and pH. For fungal communities, PGC was positively correlated with pH and negatively correlated with AP and NO_3_^−^-N. PGRC2 was positively associated with AP and SOM but negatively associated with pH and NH_4_^+^-N. PGRC3 showed positive correlations with NH_4_^+^-N and NO_3_^−^-N and negative correlations with pH and SOM. Similarly, PGCO2 was positively correlated with NO_3_^−^-N, NH_4_^+^-N, and AP, and negatively correlated with SOM and pH. PGCO3 also exhibited positive correlations with NH_4_^+^-N and NO_3_^−^-N and negative correlations with pH and SOM.

## 4. Discussion

### 4.1. Diversity of Soil Bacterial and Fungal Communities

Alpha-diversity and community structure of soil microorganisms are key indicators of soil health and ecosystem stability, as they drive biogeochemical cycles and ecosystem functioning. In ginseng cultivation systems, differences between rhizosphere and original soils can strongly influence plant growth and medicinal quality [38]. However, the combined effects of planting duration and soil compartment on microbial diversity remain unclear. Therefore, we examined changes in bacterial and fungal alpha-diversity in rhizosphere and original soils after two and three years of ginseng cultivation.

The Chao1 index of bacterial communities was higher at two years than at three years in both soil compartments (Figure 1 and Figure 2), indicating a decline in bacterial richness over time. Prolonged cultivation likely alters the soil microenvironment, leading to the loss of taxa poorly adapted to the changing conditions. Additionally, extended planting may also cause nutrient imbalance, soil structural degradation, and pathogen accumulation, collectively disrupting microbial habitats [39,40].

With increasing cultivation duration, (SOM, TN, AN) and pH significantly decreased in the rhizosphere. These changes favored oligotrophic bacteria over eutrophic groups. Soil acidification also promoted phenolic acid–producing fungi such as *Pseudogymnoascus*, whose metabolites can further acidify the soil and suppress beneficial bacteria, creating a self-reinforcing cycle of “nutrient depletion–acidification–microecological degradation” [41]. In addition, interspecific competition and ecological succession within bacterial communities may have reduced diversity, as less competitive taxes were gradually excluded.

NMDS analysis showed clear separation of microbial communities between two- and three-year systems in both soil compartments (Figure 3), demonstrating the significant impact of planting duration. These differences likely reflect dynamic interactions between ginseng roots and soil microbes. Over time, changes in the quantity and composition of root exudates influence microbial community assembly and nutrient availability, thereby affecting microbial survival and growth [42]. The rhizosphere, a hotspot of plant–microbe interactions, showed particularly pronounced restructuring with extended cultivation, including species turnover, abundance shifts, and altered microbial network relationships [43,44].

### 4.2. Composition of Soil Bacterial and Fungal Communities

Soil physicochemical properties were closely correlated with microbial abundance, indicating strong coupling between soil conditions and community structure, with consequences for soil fertility and ecosystem function. In original soil after two years of cultivation, *HSB_OF53_FO7* was the dominant taxon (Figure 4). Its abundance may reflect adaptation to local pH, moisture, and nutrient conditions, as well as an enhanced ability to utilize organic matter associated with ginseng cultivation [45]. It may also have formed synergistic interactions or competitively excluded other taxa, thereby stabilizing its niche [46]. Favorable soil structure and aeration, together with strong stress tolerance, likely supported its persistence under fluctuating conditions.

In contrast, *Tetracladium* peaked in abundance in the rhizosphere soil of three-year ginseng (Figure 4). This pattern likely reflects adaptation to rhizosphere conditions that develop over time, including shifts in pH, moisture, and nutrient accumulation. *Tetracladium* efficiently utilizes organic compounds derived from prolonged root exudation and ginseng residues [47,48]. Its competitive advantage may result from antimicrobial production and effective niche occupation. Progressive changes in soil structure and intensified rhizosphere effects likely further promoted its proliferation.

*Paraburkholderia* and *Cladophialophora* were consistently more abundant in the rhizosphere than in original soils, regardless of planting duration (Figure 5). Their enrichment suggests strong resilience and adaptability to environmental fluctuations [49,50,51]. Both genera exhibit high physiological plasticity, enabling rapid adjustment to changes in nutrient availability and pH. They maintain metabolic activity under stress—such as nutrient limitation or abrupt pH shifts—through modifications in membrane function, metabolic pathways, and stress-responsive gene expression. These traits support long-term persistence and competitive dominance in dynamic rhizosphere environments.

### 4.3. Factors Influencing Soil Microbial Communities

Correlation heatmaps showed significant positive associations between *Candidatus*_Udaeobacter and *Gemmatimonas* and soil NH_4_^+^-N content (Figure 6), indicating roles in nitrogen cycling. *Candidatus_Udaeobacter* participates in ammonification, converting organic nitrogen into NH_4_^+^-N and increasing ammonium availability in soil [52]. *Gemmatimonas* efficiently utilizes NH_4_^+^-N as a nitrogen source, providing a competitive advantage in ammonium-enriched soils [53]. It may also interact with ammonifying microorganisms, further enhancing its abundance under high-NH_4_^+^-N conditions. Together, these taxes contribute to soil nitrogen turnover and fertility.

Among fungi, *Acremonium* and *Solicoccozyma* were positively correlated with NH_4_^+^-N [54,55]. Acremonium preferentially uses NH_4_^+^-N for growth and responds sensitively to ammonium availability. *Solicoccozyma* is likely to contribute to nitrogen transformation through organic matter decomposition and enzymatic release of NH_4_^+^-N, thereby sustaining its growth in ammonium-rich soils.

Mantel analysis indicated that bacterial Chao1 richness was strongly correlated with SWC and NH_4_^+^-N, whereas fungal community composition was primarily influenced by NH_4_^+^-N (Figure 7). The Chao1 index, which estimates species richness and community complexity [56,57], increased with soil moisture, likely because adequate water enhances bacterial metabolic activity and dispersal [58]. Ammonium availability directly regulates bacterial growth and diversity. Fungal community assembly was also shaped by NH_4_^+^-N, as taxa differ in nitrogen preferences, leading to compositional shifts across nitrogen regimes.

**Figure 8:**
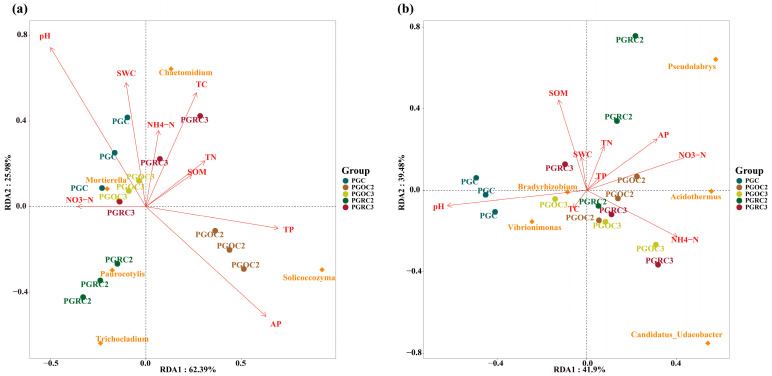
RDA analysis of the relationship between soil physico-chemical properties and the top ranked bacterial (**a**) and fungal (**b**) genera under different treatments. Different colored circles represent different treatments. Yellow squares represent different bacterial phyla. Arrows represent different soil physical and chemical property indicators. The length of the arrows represents the intensity of the influence of that environmental factor on the change in the community, and the longer the length of the arrows, the greater the influence of the environmental factor. The angle between the arrows and the axes represents the correlation between the environmental factor and the axes, the smaller the angle, the higher the correlation. The vertical distance from the sample point to the extremely extended line of the arrow of the environmental factor indicates the strength of the influence of the environmental factor on the sample, the closer the sample point is to the arrow, the stronger the effect of the environmental factor on the sample. If the sample is located in the same direction of the arrow, it means that the environmental factor is positively correlated with the change in the sample species community, and if the sample is located in the opposite direction of the arrow, it means that the environmental factor is negatively correlated with the change in the sample species community.

RDA analysis showed that *Chaetomium* was positively correlated with NH_4_^+^-N and TC and was well adapted to the rhizosphere of three-year ginseng (Figure 8). Its enrichment may result from efficient utilization of carbon and nitrogen derived from root exudates and plant residues [59,60]. It may also form mutualistic associations with ginseng, promoting plant growth while suppressing pathogenic microorganisms.

*Vibrionimonas* was positively correlated with soil pH and was primarily associated with the original soil after three years (Figure 8). This genus appears adapted to neutral or slightly alkaline conditions, which enhance nutrient availability and metabolic activity [61,62]. Prolonged cultivation likely increases organic matter and root-derived inputs in the original soil, providing a stable nutrient source that supports its persistence in mature soils.

## 5. Conclusions

This study revealed significant differences in key soil physicochemical properties (e.g., pH, AP, TP, and NO_3_^−^-N) were observed between rhizosphere and bulk soils during ginseng cultivation. Microbial community structure and diversity also shifted dynamically across cultivation years. Specifically, in the second year, bacterial alpha diversity (Chao1 and Shannon indices) was highest in bulk soil, and bacterial community composition was similar among second-year bulk soil and rhizosphere soils from both the second and third years. Concurrently, the bacterial genus *HSB_OF53_FO7* was most enriched in second-year bulk soil. By the third year, the fungal genus *Mortierella* became significantly enriched in the rhizosphere. *Chaetomium* also showed strong adaptation to the third- year rhizosphere, with positive correlations to NH_4_^+^-N and TC. Cross-year analysis revealed that the bacterial genus *Paraburkholderia* and the fungal genus *Cladophialophora* were consistently and significantly enriched in rhizosphere soil throughout cultivation. Correlation analysis indicated that bacterial diversity (Chao1 index) was strongly associated with soil water content and NH_4_^+^-N, whereas fungal community structure was primarily driven by NH_4_^+^-N. In addition, the bacterial genus *Vibrio* was positively correlated with soil pH, suggesting a preference for third-year bulk soil conditions. Collectively, these findings clarify how cultivation duration alters soil physicochemical properties and shapes microbial community dynamics. This understanding provides a theoretical basis for developing microbe-based management strategies to support sustainable ginseng production.

## Data Availability

The authors confirm that the data supporting the findings of this study are available within the article and its Supplementary Materials.

## Supplementary Materials

The following supporting information can be downloaded at: https://www.mdpi.com/article/10.3390/microorganisms14040764/s1, Table S1. Soil physical and chemical properties; Table S2. Alpha-diversity of soil bacterial communities under different treatments; Table S3. Alpha-diversity of soil fungal communities under different treatments; Figure S1a Rarefaction curve of bacteria; Figure S1b Rarefaction curve of fungi; Figure S2a Heat trees display the mean proportion of bacteria; Figure S2b Heat trees display the mean proportion of fungi.
