# Supplementary material for: Shifts in Rhizosphere and Bulk Soil Microbial Communities During the Second and Third Years of Ginseng Cultivation
Source: Microorganisms. 2026 Mar 27;14(4):764. doi: 10.3390/microorganisms14040764 (PMC13119511; doi:10.3390/microorganisms14040764)
Supplement: Supplementary file 1 [file microorganisms-14-00764-s001.zip › microorganisms-4155397-supplementary.pdf]

Table S1. Soil physical and chemical properties.

	PGC	PGOC2	PGOC3	PGRC2	PGRC3
pH	5.9±0.08a	4.8±0.1c	5.4±0.1b	5.1±0.1bc	5.5±0.08b
SWC (%)	0.2±0.01a	0.1±0.02b	0.1±0.01b	0.1±0.03b	0.2±0.01a
SOM (g/kg)	65.66±1.6a	66.0±10.2a	50.5±14.7c	63.7±12.3b	67.0±3a
TC (%)	3.3±0.1b	2.9±0.5c	2.4±0.54d	2.7±0.2cd	3.6±1a
TP (mg/kg)	989.6±65.08b	1464.8±227.8a	616.7±31.4d	796.2±126.9c	899.3±48.2b
AP (mg/kg)	41.7±3.8c	78±1.8a	38.3±1.6c	54.6±9.0b	35.7±6.3c
TN (%)	0.3±0.01a	0.35±0.04a	0.3±0.1a	0.3±0.08a	0.3±0.1a
NO_3^--N (mg/kg)	7.9±0.9d	2.1±0.1d	64.2±4.9b	79±8.9a	89.0±3.5a
NH_4^--N (mg/kg)	61.5±3.2e	70.9±5.08d	99.7±0.05b	82.5±3.4c	156.3±7.5a

Note: Potential of hydrogen; SWC: Soil water content; SOM: Soil organic matter; TC: Total carbon; TP: Total Phosphorus; AP: Available Phosphorus; TN: Total nitrogen; NO_3^--N: nitrate nitrogen; NH_4^+-N: ammonium nitrogen.

Table S2. Alpha-diversity of soil bacterial communities under different treatments.

	Richness	ACE	Simpson	Pielou
PGC	329.3±53.7b	334.5±58.5b	0.9±0.005a	0.7±0.01a
PGOC2	381. 7±33.3a	385.1±34.9a	0.9±0.001a	0.7±0.01a
PGOC3	310. 7±76.5c	313.2±77.2c	0.9±0.009a	0.7±0.02a
PGRC2	315.0±35.5c	317.6±34.8c	0.9±0.002a	0.7±0.008a
PGRC3	304.0±13.1c	309.8±9.5c	0.9±0.003a	0.7±0.02a

Table S3. Alpha-diversity of soil fungal communities under different treatments.

	Richness	ACE	Simpson	Pielou
PGC	171.3±16.2a	171.6±16.6b	0.9±0.005a	0.7±0.02a
PGOC2	179.0±19.1a	180.0±19.9a	0.9±0.01a	0.6±0.02b
PGOC3	166.3±9.6b	166.7±9.9b	0.9±0.006a	0.6±0.02b
PGRC2	131.3±22.3c	131.5±22.5d	0.9±0.02a	0.6±0.02b
PGRC3	153.6±11.3b	154.0±11.6c	0.9±0.01a	0.6±0.02b

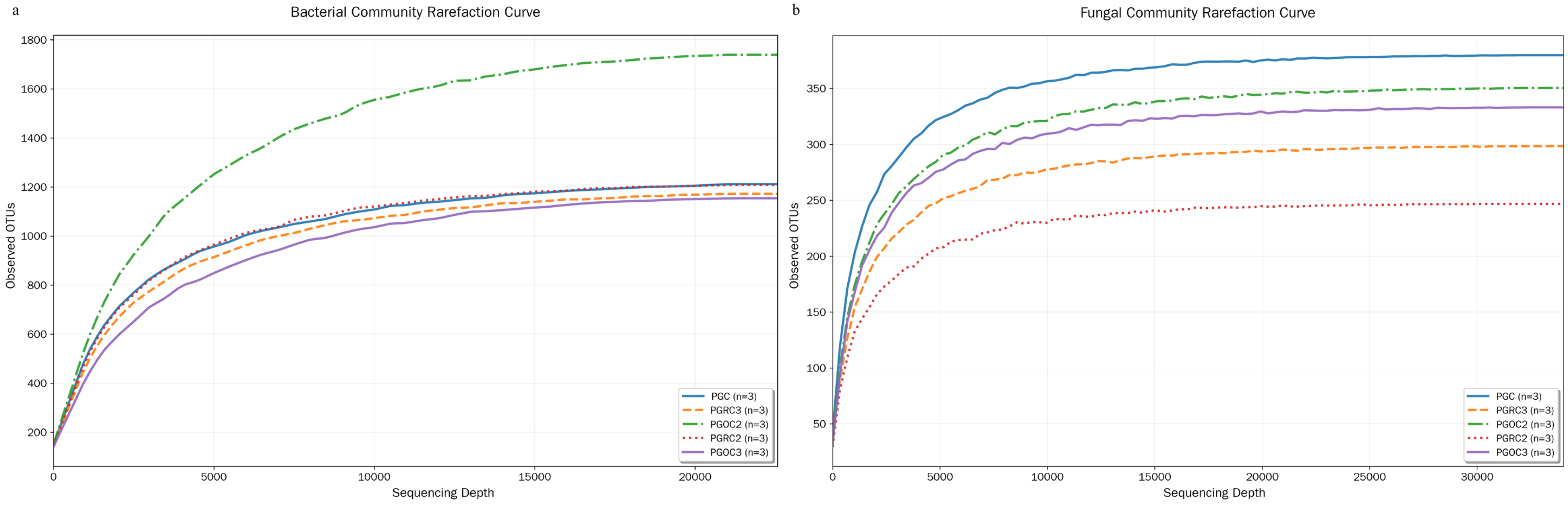

Figure S1. Rarefaction curve of bacteria (a); Rarefaction curve of fungi (b)

(a) **(b)**

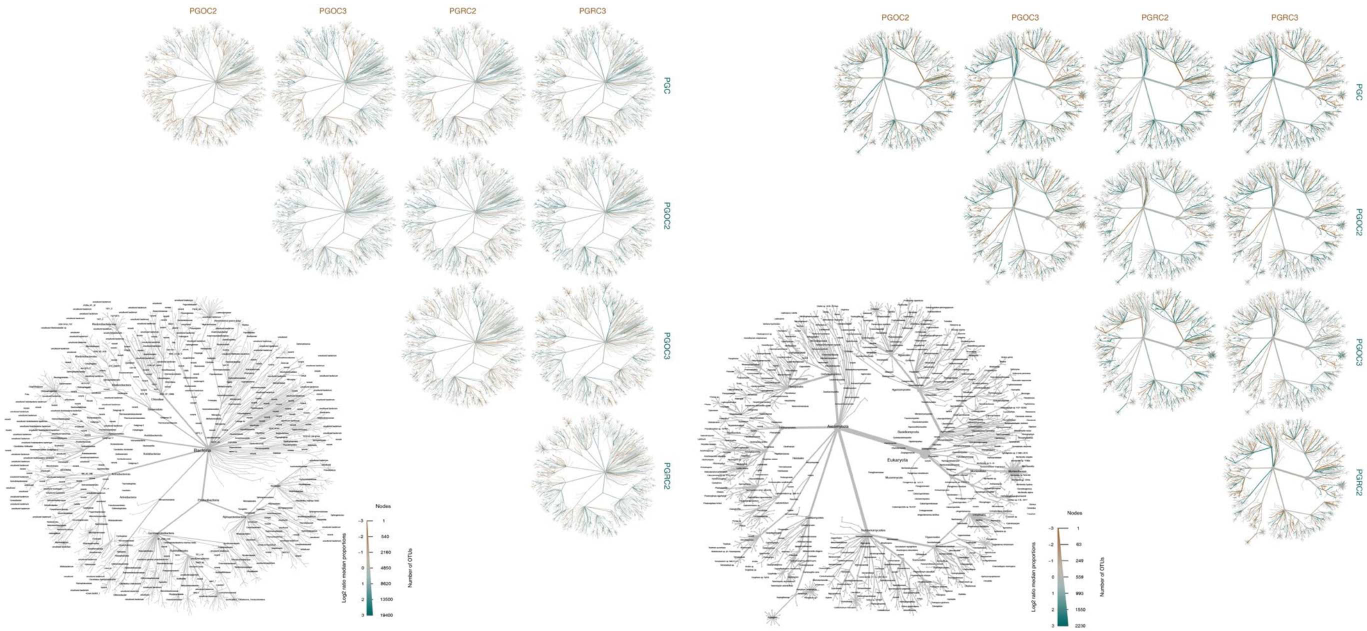

Figure S2. Heat trees display the mean proportion of bacteria(a) and fungi(b) in different treatments. Nodes represent each taxonomic level from kingdom (bacteria, center) to species (top of each branch). The width of nodes and edges (branches) represents the average proportion of samples belonging to this classification group. The size of the node corresponds to the number of taxonomic groups, and the depth of the color corresponds to the proportion of the bacterial sample population. The color of each taxonomic group represents the log-2 ratio of the median proportion of readings observed in each body. Using Wilcox rank sum test and Benjamini and Hochberg (FDR) multiple comparison correction method, only significant differences will be colored. The gray tree in the lower left corner is the key to smaller unmarked trees. The green classification group is enriched in the microbial soil portion shown in this row, while the brown classification group is enriched in the microbial portion shown in this column.
